# Molecular disruption of NBS1 with targeted gene delivery enhances chemosensitisation in head and neck cancer

**DOI:** 10.1038/sj.bjc.6605980

**Published:** 2010-11-09

**Authors:** K Araki, T Yamashita, N Reddy, H Wang, W M Abuzeid, K Khan, B W O'Malley, D Li

**Affiliations:** 1 Department of Otorhinolaryngology-Head & Neck Surgery, University of Pennsylvania School of Medicine, 415 Curie Boulevard, CRB Room 145, Philadelphia, PA 19104, USA; 2 Department of Otorhinolaryngology-Head & Neck Surgery, National Defense Medical College, 3-2 Namiki, Tokorozawa, Saitama 359-8513, Japan

**Keywords:** chemoresistance, NBS1, adenovirus, targeted gene therapy, DNA double-strand breaks, DNA repair

## Abstract

**Background:**

A fibroblast growth factor 2 (FGF2)-targeted adenoviral system can alter viral tropism and allow for improved transduction and reduced systemic toxicity. This study is to investigate if the FGF2-targeted adenoviral mutant Nijmegen breakage syndrome 1 (FGF2-Ad-NBS1) gene transfer can enhance cisplatin chemosensitisation not only by targeting DNA repair, but also through the induction of antiangiogenesis, whereas at the same time reducing toxicities in treating head and neck squamous cell carcinoma (HNSCC).

**Methods:**

The human HNSCC cell line was treated *in vitro* and in a nude mouse xenograft model. We conducted verification of binding ability of mutant NBS1 and downregulation of MRN complex, evaluation of transduction efficiency and combined antitumour activities. The antiangiogenesis mechanism was also investigated. Finally, we estimated the distribution of adenoviral vector in the liver.

**Results:**

The mutant NBS1 protein retains the binding ability and effectively suppresses the expression level of the MRN in infected cells. Transduction efficiency *in vitro* and cisplatin chemosensitisation were upregulated. The FGF2-Ad-NBS1 also showed detargeting the viral vectors away from the liver. The downregulation of NF-*κ*B expression was supposed to correlate with increased antiangiogenesis.

**Conclusions:**

FGF2-targeted adenoviral system enhances the cisplatin chemosensitisation of mutant NBS1 and may avoid viral-associated liver toxicities.

Cisplatin is one of the most commonly used agents for the treatment of the head and neck squamous cell carcinoma (HNSCC). Cisplatin cytotoxicity results from the formation of DNA monoadducts and crosslinks, which, in turn, promote the development of apoptosis-inducing double-strand breaks (DSBs) during replication (Chu, 1994; Perez, 1998). Unfortunately, cisplatin-based tumour resistance is often acquired after repeated cycles of therapy or owing to inherent resistance to cisplatin in clinical practice. The development of resistance to cisplatin is considered one of the major factors of treatment failure in HNSCC (Kelland, 2007). A major factor in the development of chemoresistance is the enhanced DNA repair ability of tumour cells preventing the accumulation of lethal DNA damage from cytotoxic agents (Drummond *et al*, 1996; Fink *et al*, 1996; Perez, 1998; Kartalou and Essigmann, 2001; Martin *et al*, 2008). Therefore, interruption of cellular DNA repair mechanisms can sensitise tumour cells to DNA-damaging agents (O'Malley *et al*, 2003; Tran *et al*, 2004; Abuzeid *et al*, 2009).

One of the critical components in the DNA DSB repair system is the protein complex made up of the MRE11, RAD50 and NBS1 proteins (MRN complex) (Williams *et al*, 2007). Mutations in the *NBS1* gene that codes for the NBS1 protein cause the congenital disorder Nijmegen breakage syndrome (NBS). Nijmegen breakage syndrome 1 participates in the recognition of DNA damage and subsequent recruitment of MRE11 and RAD50, forming DNA repair complexes at the sites of DSB damage (Kobayashi *et al*, 2004). Patients with NBS are characterised by stunted growth, microcephaly, marked sensitivity to ionising radiation, chromosome translocations, and increased incidence of leukaemia and lymphoma. The pathogenesis of NBS arises from premature truncation of the protein product (Varon *et al*, 1998). This disorder underscores the fact that disruption of MRN function leads to a dysfunction of DNA DSB repair system and consequently sensitises tumour cells to DNA damage agents, including cisplatin. We have developed an adenoviral vector containing mutant *NBS1* (Ad-NBS1) or *RAD50* (Ad-RAD50) for the purpose of disrupting MRN function and sensitising cells for cisplatin. In human HNSCC cell lines *in vitro*, Ad-NBS1 gene transfer significantly increased cisplatin-induced DNA DSBs, cytotoxicity and inhibited tumour cell growth (Tran *et al*, 2004). Also, it has shown a significant antitumour effect after combination with radiation therapy (O'Malley *et al*, 2003). A combination of cisplatin and mutant RAD50 therapy also produced significant tumour cytotoxicity *in vitro*, with a corresponding increase in DNA damage and telomere shortening. In cisplatin-resistant human HNSCC xenografts in nude mice, this combination therapy caused dramatic tumour regression with increased apoptosis (Abuzeid *et al*, 2009).

Head and neck squamous cell carcinomas are suitable for local viral vector delivery, because most of them are locally accessible and can be directly approached in our clinical practice. Adenoviral transduction efficiency in most cells is highly dependent on the expression of its primary receptor, coxsackie virus-adenovirus receptor (CAR) (Li *et al*, 1999). Low and varied expression of CAR could be a significant factor for low transduction efficiencies. Several studies have investigated the benefit of adenoviral targeting from its tropism for CAR to the fibroblast growth factor receptors (FGFRs), which significantly increases adenoviral gene transduction to FGFR-positive cells, and which are less prevalently expressed on non-cancerous tissues (Goldman *et al*, 1997; Rogers *et al*, 1997; Gu *et al*, 1999). Fibroblast growth factor 2 (FGF2)-targeted adenoviral conjugates (FGF2-Ad) utilises FGF2 as the targeting ligand. This FGF2-targeted adenoviral system has achieved efficient gene transfer in previously reported ovarian, pancreatic, skin and lung cancer models, as well as in HNSCC and in endothelial cells (ECs) (Rogers *et al*, 1997; Rancourt *et al*, 1998; Kleeff *et al*, 2002; Qin *et al*, 2005; Saito *et al*, 2009). Fibroblast growth factor 2 exhibits its biological activities by high affinity binding to FGFRs (particularly FGFR1 and FGFR2). The expression levels of FGFR1 and FGFR2 are known to be upregulated in cancer cells and ECs in HNSCC (Dellacono *et al*, 1997; Wakulich *et al*, 2002). Given the relationship between FGF2 expression and ECs, and the low level of CAR expression on ECs in HNSCC, there may be a potential role for FGF2-targeted therapy as an additional therapeutic strategy of antiangiogenesis.

Nuclear factor-*κ*B is a well-known transcription factor that is responsible for regulating many genes involved, such as immune response differentiation, proliferation, angiogenesis and apoptosis (Sun and Zhang, 2007). It is regulated by the Ras/MEK signalling pathway, which is parallel with PI3K/AKT angiogenesis pathway (Jiang and Liu, 2008). Therefore, the expression level of NF-*κ*B may vary from the status of angiogenesis in tumour.

In this study, we first evaluated if disruption of MRN function by Ad-NBS1 sensitised HNSCC to cisplatin *in vivo*. Next, we examined if Ad-NBS1 specifically targeting FGFRs could enhance the therapeutic benefit with cisplatin treatment through not only targeting cancer cells, but also disrupting angiogenesis. The status and mechanism of antiangiogenesis after FGF2-targeted Ad-NBS1 delivery and the possibility of systemic toxicity reduction by altering organ distribution were also investigated.

## Materials and methods

### Cell line

The human HNSCC cell line JHU006 (originally derived at the Johns Hopkins University Head and Neck Laboratories, Baltimore, MD, USA) was used in all experiments. This cell line has derived from human tumour explants, has been well characterised and is known to express wild-type MRE11, RAD50, NBS1, high FGFRs and low CAR. The 50% inhibitory concentration (IC_50_) of 72 h after cisplatin administration for this cell line is 2.6 *μ*g ml^−1^. Cells were propagated as described previously (Li *et al*, 2002).

### Animals

Animal experiments were performed on 6-week-old BALB/c nude mice maintained in an animal facility. Animals were cared for and used in accordance with protocols approved by the Animal Care and Use Committee of The University of Pennsylvania.

### Recombinant adenoviral vector and FGF2-Fab′ adenovirus conjugate

An adenovirus construct containing the mutant NBS1 cDNA for the C-terminal 300 amino acids of NBS1 and green fluorescent protein (GFP) under the control of two independent cytomegalovirus (CMV) promoters (Ad-NBS1) was created (Li *et al*, 1999). Green fluorescent protein-expressing adenovirus under the control of CMV promoters (Ad-GFP) was used for control virus. A bi-functional FGF2-Fab′conjugate molecule was prepared based on published protocols (Doukas *et al*, 1999) and obtained from Selective Genetics Inc. (San Diego, CA, USA).

### Western blot analysis

The expression of wild-type MRE11, RAD50, NBS1 and mutant NBS1 proteins was detected using western blot analysis as described previously (Tran *et al*, 2004; Abuzeid *et al*, 2009). Recombinant adenovirus (Ad-NBS1 or Ad-GFP) was introduced into JHU006 cell cultures at a multiplicity of infection (MOI) of 10 for 4 h at 37°C. Polyclonal rabbit anti-human MRE11, RAD50, NBS1, which recognises both wild-type and mutant NBS1, and *β*-actin (Novus Biologicals, Littleton, CO, USA) was used as the primary antibody at a concentration of 1 : 1000. MagicMark XP western protein standard (Invitrogen Corporation, Carlsbad, CA, USA) was used to monitor electrophoresis progression. Western Breeze chemiluminescent immunodetection system (Invitrogen) was used to observe protein bands. Experiments were repeated in triplicate. Images of the protein bands were acquired using a Canon Digital Rebel XTI camera (Canon USA Inc., New York, NY, USA). Densitometric analysis of the films was performed using the ImageJ 1.43 software, which is publically available through the National Institute of Health.

The expression of NF-*κ*B protein after FGF2-targeted NBS1 gene delivery *in vitro* was also detected using western blot analysis, same as described above. Cultured JHU006 cells were split into five groups: mock-treated PBS as ‘Control’, Ad-GFP (Control virus), Ad-NBS1, FGF2-Fab′-conjugated Ad-GFP (FGF2-Ad-GFP) and FGF2-Ad-NBS1. Recombinant adenovirus- or FGF2-Fab′-conjugated adenovirus was introduced at an MOI of 5 for 4 h at 37°C and then cultured for 72 h. Polyclonal rabbit anti-human NF-*κ*B (Cell Signaling Technology, Danvers, MA, USA) and polyclonal rabbit anti-*β*-actin (Novus Biologicals, Littleton, CO, USA) were used at a concentration of 1 : 1000 as primary antibodies. Densitometric analysis of the films was performed using the ImageJ 1.43 software, which is publically available through the National Institute of Health. Experiments were repeated in triplicate.

### Co-immunoprecipitation

Co-immunoprecipitation (Co-IP) was conducted to verify the structural integrity between MRE11 and NBS1 following Ad-NBS1 infection in tumour cells as described previously (Abuzeid *et al*, 2009). JHU006 cells were either untreated or infected with Ad-NBS1 or Ad-GFP at an MOI of 10 for 24 h. Monoclonal mouse anti-human MRE11 antibody (Novus Biologicals, Littleton, CO, USA) was used to pull down the components of the MRN complex at a concentration of 1 : 1000. The immunoprecipitates were analysed by western blot analysis.

### Transduction efficiency and tumour cell growth *in vitro*

To investigate the transduction efficiency and cisplatin chemosensitisation effect after FGF2-targeted adenoviral gene delivery, 3 × 10^3^ JHU006 cells were plated in 96-well tissue culture plates. Wells were split into eight groups: mock-treated PBS as ‘Control’, cisplatin, Ad-NBS1, Ad-NBS1/cisplatin, FGF2-Fab′-conjugated Ad-NBS1 (FGF2-Ad-NBS1) and FGF2-Ad-NBS1/cisplatin. Cells were incubated with any of the adenovirus or PBS at an MOI of 5 or 10. After 24 h, cisplatin was added at a concentration of 0.3 *μ*g ml^−1^. The transduction efficiencies of Ad-NBS1 and FGF2-Ad-NBS1 *in vitro* were evaluated by GFP expression at 1 and 3 days after treatment. Views of each well were digitally recorded at 100-fold magnification under fluorescent microscopy (Eclipse TS100, Nikon, Tokyo, Japan). The average intensity of GFP expression was obtained with the assistance of IP Lab software (Scanalytics Inc., Fairfax, VA, USA).

The tumour cell growth was evaluated by MTT assay for 6 consecutive days (day 0–5) as described previously (Araki *et al*, 2008). The optical density was determined by spectrophotometry (MRX II, Dynex Technologies, Chantilly, VA, USA).

### Xenograft tumours *in vivo*

Tumours were established in the right flank of nude mice by a subcutaneous (s.c.) injection of 1.0 × 10^7^ JHU006 cells. At 14 days after tumour injection, tumours were surgically exposed and measured in three dimensions. Subsequently, intratumoral injections of 50 *μ*l of adenoviral vectors (3.0 × 10^8^ PFU ml^−1^) or PBS were carried out using a Hamilton syringe. Cisplatin was intraperitoneally injected in cisplatin-treated groups at 5 mg kg^−1^ as a regular dose or 3 mg kg^−1^ as a reduced dose. Tumour masses were measured again in three dimensions and harvested at the time of termination.

For the first trial, to investigate the cisplatin chemosensitisation effects after Ad-NBS1 transfer, 30 mice were randomly divided into six groups: PBS as ‘Control’, Reduced dose of cisplatin (Reduced cisplatin), Regular dose of cisplatin (Regular cisplatin), Ad-NBS1, Ad-NBS1/Reduced cisplatin and Ad-NBS1/Regular cisplatin. The second trial was performed to investigate the advantage in cisplatin chemosensitisation after FGF2-targeted Ad-NBS1 therapy. A total of 25 mice were randomly divided into five groups: PBS, Regular cisplatin, Ad-NBS1/Reduced cisplatin, Ad-NBS1/Regular cisplatin and FGF2-Ad-NBS1/Reduced cisplatin. The tumour volume was measured 17 days after treatments, apoptosis induction was evaluated in both first and second trials and angiogenesis was evaluated in the second trials.

To confirm the potential reduction of systemic side effects and to detect the mechanism of the antiangiogenesis effect, the third trial was performed. In total, 60 mice were randomly divided into three groups: PBS, Ad-NBS1 and FGF2-Ad-NBS1. Five mice of each group were euthanised at 1, 3, 7 and 14 days after viral injection, and tumours and livers were harvested. The existence of adenovirus in the liver was investigated by polymerase chain reaction (PCR).

### Apoptosis detection

The ApopTag Peroxidase *In-Situ* Apoptosis Detection Kit (Millipore, Billerica, MA, USA) was used to detect apoptosis. The staining was performed following the manufacturer's protocol and slides were viewed under the microscope (Eclipse 80i, Nikon, Tokyo, Japan). Four randomly selected views at 200-fold magnification were digitally recorded in each tumour. All positive cells within the view were counted.

### Immunohistochemistry

Immunohistochemistry was performed using rat monoclonal anti-CD31 (PECAM-1) antibody (BD PharMingen, San Diego, CA, USA) and the VECTASTAIN *Elite* ABC kit (Vector Laboratories Inc., Burlingame, CA, USA) to visualise ECs (CD31) of the second trial. To assess microvessel growth, four views ( × 200) were digitally recorded for each tumour to cover the greatest anti-CD31 staining area for the estimation of microvessel development (Weidner, 1995). The relative percentage of CD31 staining positive area (% region of interest: %ROI), which represents the microvessel density (MVD), within each view was measured with the assistance of IP Lab software.

### Virus detection in the liver

Total DNA was isolated from the tissues of harvested livers using DNeasy, Blood & Tissue Kit (Qiagen Science, Germantown, MD, USA) in accordance with the instructions. Polymerase chain reaction was performed in PCR buffer. Specific oligonucleotide primers were designed to amplify Ad-NBS1 gene-specific derived DNA (forward, 5′-TTGACGCAAATGGGCGGTAGG-3′ reverse, 5′-CTGCAGCATGAGATTTACTGG-3′), which produces a 381-bp amplified product. The PCR amplification program comprises 35 cycles of: denaturation at 94°C for 45 s, annealing at 60°C for 45 s and extension at 72°C for 45 s.

### Statistical analysis

Mann–Whitney analysis was applied using STATMOST (Detaxion Software Inc., Los Angels, CA, USA) to determine the statistical significance.

## Results

### Transgene expression of mutant NBS1 disrupts the MRN functional unit

**Figure 1:**
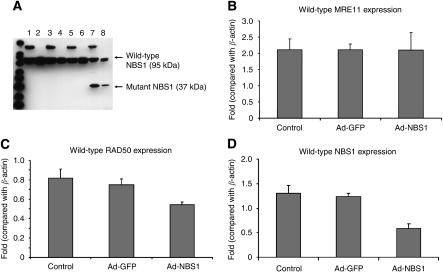
Transgene expression of mutant NBS1 interacts with MRN after Ad-NBS1 gene transfer and significantly reduces the expression of wild-type RAD50 and NBS1. (**A**) Anti-MRE11 antibody Co-IP wild-type NBS1 in all cells. It indicated direct interaction between MRE11 and wild-type NBS1 (lanes 2, 4, 6 and 8). Mutant NBS1 was Co-IP in Ad-NBS1-infected cells, indicated direct interaction between MRE11 and mutant NBS1 (lane 8). A significant downregulation of wild-type NBS1 protein was observed in cells infected with Ad-NBS1 (lane 8) relative to non-infected cells (lanes 2, 4 and 6). All lanes shown were run simultaneously on a single gel as contiguous lanes. Lane 1: control cells; lane 2: control cells after Co-IP with MRE11 antibody; lane 3: cells treated with cisplatin (0.3 *μ*g ml^−1^); lane 4: cisplatin-treated cells after Co-IP with MRE11antibody; lane 5: Ad-GFP-infected cells; lane 6: Ad-GFP-infected cells after Co-IP; lane 7: Ad-NBS1-infected cells and lane 8: Ad-NBS1-infected cells after Co-IP with MRE11 antibody. (**B**–**D**) Wild-type MRE11 was not downregulated after infection of JHU006 cells with Ad-NBS1 relative to non-infected controls or Ad-GFP controls (**B**). Downregulation of wild-type RAD50 (*P*<0.05; **C**) and NBS1 proteins (*P*<0.01; **D**) is seen after infection with Ad-NBS1 when compared with non-infected controls or Ad-GFP controls.

Co-immunoprecipitation was used to confirm that MRE11 forms complex with wild-type NBS1 or mutant NBS1. In non-infected cells or Ad-GFP-infected cells, anti-MRE11 antibody co-precipitated NBS1 (95 kDa; Figure 1A). After Ad-NBS1 infection, anti-MRE11 antibody was able to co-precipitate both NBS1 and mutant NBS1 (37 kDa; Figure 1A). These findings indicate that both wild-type and mutant NBS1 protein retain binding ability to the MRE11/RAD50 complex. Western blot after Co-IP showed that there was a significant downregulation of wild-type NBS1 protein in cells infected with Ad-NBS1 relative to non-infected cells (Figure 1A). These results showed that Ad-NBS1 effectively suppresses the expression of the MRN functional unit in infected cells.

### MRN protein expression in Ad-NBS1-infected or -uninfected JHU006 cells

We confirmed the expression of wild-type MRE11 (81 kDa; Figure 1B), RAD50 (153 kDa; Figure 1C) and NBS1 (Figure 1D) proteins in JHU006 cells by western blot. The wild-type MRE11 proteins expression showed no significant differences between uninfected and Ad-NBS1-infected JHU006 (Figure 1B). The expressions of wild-type RAD50 (Figure 1C; *P*<0.05) and NBS1 (Figure 1D; *P*<0.01) were significantly reduced in Ad-NBS1-infected JHU006 cells when compared with Ad-NBS1-uninfected cells. Adenoviral-green fluorescent protein did not show significant difference when compared with control. It means that GFP or CMV promoter does not affect the expression of MRN complex in this cancer cells. Mutant NBS1 protein expression was observed only in Ad-NBS1-infected JHU006 cells.

### FGF2-targeted adenoviral system enhances transduction efficiency *in vitro*

**Figure 2:**
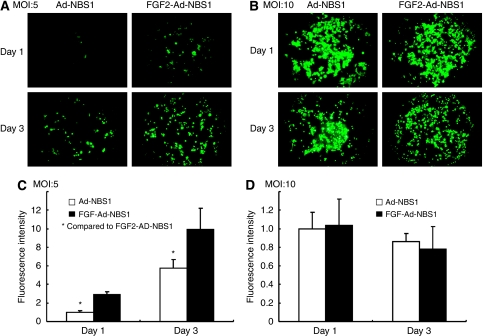
Fibroblast growth factor 2-targeted gene delivery system significantly increases transgene expression in tumour cells. Representative pictures after 1 and 3 days after adenoviral infection at an MOI of 5 (**A**) and 10 (**B**). Relative intensity of GFP expression at an MOI of 5 (**C**) and 10 (**D**). The benefit of FGF2-targeted transgene was greater at an MOI of 5 and sufficient gene transduction looks promising even after a lower titre adenoviral administration. ^*^P<0.01.

We investigated the benefit of FGF2-targeted adenoviral system in gene transduction. The average relative intensity of GFP expression after FGF2-targeted Ad-NBS1 and non-targeted Ad-NBS1 gene transfer in the JHU006 cell line at MOIs 5 and 10 was estimated at days 1 and 3 (Figure 2A and B). A significantly higher intensity was observed at both days 1 and 3 in FGF2-Ad-NBS1 than in Ad-NBS1 (2.89±0.34 *vs* 1.00±0.14, *P*<0.01, 9.90±2.28 *vs* 5.73±0.95, *P*<0.01, respectively) at an MOI of 5 (Figure 2C). In contrast, no significant difference was observed at an MOI of 10 at both days 1 and 3 (1.04±0.28 *vs* 1.00±0.17, *P*<0.01, 0.78±0.24 *vs* 0.86±0.08, *P*<0.01, respectively) (Figure 2D). These results showed that the FGF2-targeting system could achieve sufficient gene transduction after a relative lower titre adenoviral administration.

### FGF2-targeted Ad-NBS1 gene transfer enhances cisplatin chemosensitisation *in vitro*

**Figure 3:**
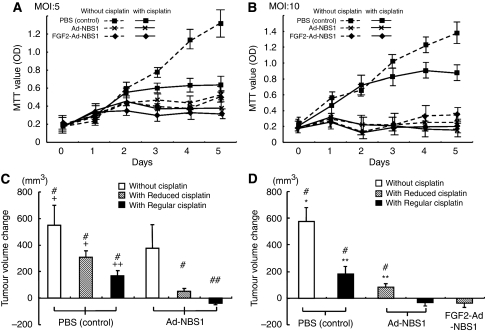
Fibroblast growth factor 2-targeted Ad-NBS1 gene transfer sensitizes head and neck cancer with cisplatin-based chemotherapy *in vitro* and *in vivo.* Tumour cell growth curve analysis *in vitro* at an MOI of 5 (**A**) and 10 (**B**) in six different treatment groups. Fibroblast growth factor 2-targeted Ad-NBS1 with cisplatin group had the greatest tumour suppression effect at an MOI of 5 (**A**), whereas no difference was observed between FGF2-Ad-NBS1/Cisplatin and Ad-NBS1/Cisplatin at an MOI of 10 (**B**). The FGF2-targeted system might enable a lower titre administration. Tumour volume changes before and after treatments are shown in the mouse model. (**C**) The greatest tumour shrinkage occurred when Ad-NBS1 gene therapy was combined with Regular cisplatin. The tumour treated with Ad-NBS1/Reduced cisplatin also showed better a tumour suppression effect when compared with the regular dose of cisplatin-treated tumours. (**D**) The combination Ad-NBS1/Regular cisplatin and FGF2-Ad-NBS1/Reduced cisplatin had the greatest efficacy in reducing tumour size and the effect was almost the same in both treatments. Fibroblast growth factor 2-Ad-NBS1 therapy enhances sensitivity for cisplatin intensely and allows for reduction of cisplatin dose. ^#^*P*<0.01 when compared with Ad-NBS1/Regular dose of cisplatin, ^##^*P*<0.05 when compared with Ad-NBS1/Regular dose of cisplatin, ^+^*P*<0.01 when compared with Ad-NBS1/Reduced dose of cisplatin, ^++^*P*<0.05 when compared with Ad-NBS1/Reduced dose of cisplatin, ^*^P<0.01 when compared with FGF2-Ad-NBS1/Reduced dose of cisplatin, ^**^P<0.05 when compared with FGF2-Ad-NBS1/Reduced dose of cisplatin.

The benefit of FGF2-targeted adenoviral system in tumour suppression was investigated using the MTT assay. JHU006 cells were subjected to six interventions. The cell growth in Ad-NBS1/cisplatin and FGF2-Ad-NBS1/cisplatin had a greater inhibition than that in other groups at an MOI of 5 (Figure 3A). The cell growth of Ad-NBS1/cisplatin and FGF2-Ad-NBS1/cisplatin also had a greater inhibition than that of the Control or Cisplatin groups at an MOI of 10 (Figure 3B); however, no difference was observed when compared with that of Ad-NBS1 or FGF2-Ad-NBS1. These results at an MOI of 5 show that the tumour inhibition in FGF2-Ad-NBS1/cisplatin has more effect at an early phase than that of Ad-NBS1/cisplatin. This is consistent with the results of the GFP expression in higher and earlier transduction of FGF2-Ad-NBS1/cisplatin than that of Ad-NBS1/cisplatin. However, the results at an MOI of 10 suggest that the transduction efficiency and tumour growth inhibition reaches the maximum level in Ad-NBS1/cisplatin at an MOI of 10. As a result, the FGF2-targeted system has the potential of a lower titre administration of virus while still achieving optimal cisplatin chemosensitisation effects.

### Transgene expression of mutant NBS1 induces cisplatin chemosensitisation in a mouse model with human HNSCC

A mouse model with JHU006 was used to evaluate whether the *in vitro* cytotoxicity caused by the disruption of MRN function translates into a beneficial effect *in vivo*. Mice were subjected to six interventions (Figure 3C).

Ad-NBS1/Regular cisplatin had the greatest efficacy in reducing tumour size. Adenoviral-NBS1/Reduced cisplatin also had better tumour suppression. No significance was observed in tumour shrinkage of Ad-NBS1/Reduced cisplatin when compared with Ad-NBS1/Regular cisplatin or PBS/Regular cisplatin. These results suggest that Ad-NBS1 induces a cisplatin chemosensitisation effect and has the potential to reduce the dose of cisplatin administration while achieving similar tumour suppressive effects of regular cisplatin without Ad-NBS1 *in vivo*.

### FGF2-targeted Ad-NBS1 gene transfer significantly enhances cisplatin chemosensitisation in a mouse model with human HNSCC

The same mouse model was used to further evaluate whether the *in vitro* transduction efficiency and cytotoxicity after FGF2-targeted Ad-NBS1 gene transfer translated into a beneficial effect on *in vivo* tumour growth. Mice were randomly divided into five groups (Figure 3D). Adenoviral-NBS1/Regular cisplatin and FGF2-Ad-NBS1/Reduced cisplatin had the greatest efficacy in shrinking tumour size and the effects were similar in both treatments. These results show that cisplatin chemosensitivity following Ad-NBS1 gene transfer is significantly enhanced by the FGF2-targeting system and allows for a reduction of cisplatin dose.

### FGF2-targeted system does not show additional benefits on Ad-NBS1-induced apoptosis *in vivo*

**Figure 4:**
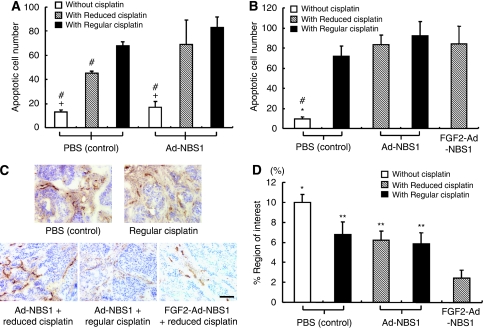
Cisplatin chemosensitisation mechanisms after FGF2-targeted Ad-NBS1 gene transfer *in vivo.* The apoptotic index was determined in the tumour samples. (**A**) The apoptotic index was found to be significantly higher in Ad-NBS1/Regular cisplatin samples. Tumours treated with Ad-NBS1/Reduced cisplatin exhibited similar levels as those tumours treated with regular dose of cisplatin. (**B**) The apoptotic index was found to be significantly higher in the all treated groups than in controls. No significant difference was found between treated groups. ^#^*P*<0.01 when compared with Ad-NBS1/Regular dose of cisplatin, ^+^*P*<0.01 when compared with Ad-NBS1/Reduced dose of cisplatin, ^*^P<0.01 when compared with FGF2-Ad-NBS1/Reduced dose of cisplatin. (**C**) Representative CD31 staining figures show significantly less staining in the tumours of FGF2-Ad-NBS1/Reduced cisplatin. (**D**) A significantly smaller %ROI CD31 staining was observed in FGF2-Ad-NBS1/Reduced cisplatin when compared with control and the other treatment groups. Scale bar: 100 *μ*m; ^*^P<0.01 when compared with FGF2-Ad-NBS1/Reduced cisplatin; ^**^P<0.05 when compared with FGF2-Ad-NBS1/Reduced cisplatin.

The number of apoptotic cells of the first animal trial was counted to evaluate the *in vivo* cisplatin chemosensitisation effect after Ad-NBS1 gene transfer (Figure 4A). The apoptotic index in the Ad-NBS1/Regular cisplatin was highest of all groups. Adenviral-NBS1/Reduced cisplatin exhibited a similar level in apoptosis induction to the regular cisplatin without Ad-NBS1.

The number of apoptotic cells of the second trial following FGF2-targeted Ad-NBS1 gene transfer was also evaluated (Figure 4B). Adenoviral-uninfected PBS control with regular cisplatin did not yield significant differences when compared with any other adenoviral-infected groups. This result indicates that apoptosis induction is not the sole factor of the enhanced cytotoxic effect after FGF2-targeted delivery.

### Antiangiogenesis is associated and enhanced with FGF2-targeted Ad-NBS1 gene transfer

The level of CD31 positivity as measured in %ROI was used to assess the level of angiogenesis in the second trial. A significantly smaller %ROI was observed in the FGF2-Ad-NBS1/Reduced cisplatin (2.40±1.78%) than all other groups (Figure 4C,D). These results indicate that a potent antiangiogenesis effect was induced after FGF2-targeted Ad-NBS1 gene transfer and this effect is postulated to be associated with enhanced cisplatin chemosensitisation.

### NF-*κ*B suppression regulates antiangiogenesis following FGF2-targeted Ad-NBS1 gene transfer

**Figure 5:**
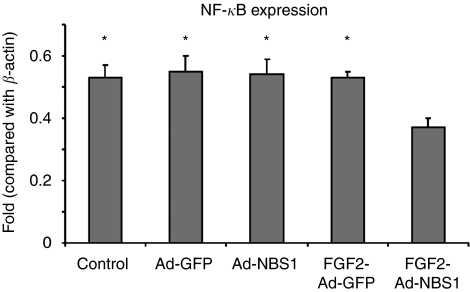
Downregulation of NF-*κ*B expression after FGF2-targeted Ad-NBS1 gene transfer. Downregulation of NF-*κ*B expression (*P*<0.05) is seen after infection with FGF2-targeted Ad-NBS1 relative to non-infected controls, Ad-GFP or Ad-NBS1. This result indicates that FGF2-targeted Ad-NBS1 downregulates NF-*κ*B activity, and which may lead to antiangiogenesis effects. ^*^P<0.01 when compared with FGF2-Ad-NBS1.

To investigate the status and mechanism of an antiangiogenesis effect after FGF2-targeted Ad-NBS1 gene transfer *in vitro*, the expression levels of NF-*κ*B proteins were analysed. NF-*κ*B is a major factor in the Ras/Raf-1 angiogenesis pathway. The expression of NF-*κ*B protein was significantly lower in FGF2-targeted Ad-NBS1-infected cells (0.37±0.03) when compared with uninfected control (0.53±0.04, *P*<0.01), Ad-GFP-infected (0.55±0.05, *P*<0.01), Ad-NBS1-infected (0.54±0.05, *P*<0.01) or FGF2-Ad-GFP-infected (0.53±0.02, *P*<0.01) cells (Figure 5). These results indicate that FGF2-targeted Ad-NBS1 may downregulate NF-*κ*B activity and downregulation of NF-*κ*B may result in antiangiogenesis in cancer cells.

### FGF2-targeted gene delivery system alters adenovirus distribution and detargets viral vectors from the liver

Viral toxicity in the liver has been a major concern following adenoviral gene transfer. To investigate if the FGF2-targeted system can alter the distribution of virus in the liver, the existence of adenoviral vector in the liver after Ad-NBS1 treatment was investigated by PCR. The adenoviral vector was detected as positive bands only in the livers of the Ad-NBS1 group (Table 1). No positive bands were found in the livers of control and FGF2-Ad-NBS1 (Table 1). Fibroblast growth factor 2-targeted system can alter the tropism of the virus, prevent the localisation of adenovirus in the liver and may have the potential to reduce systemic side effects.

## Discussion

This study showed that mutant NBS1 transfer significantly increased chemosensitivity to cisplatin *in vivo*. These effects are most likely due to molecular disruption of the MRN complex through the expression of mutant NBS1. The mutant NBS1 used in this study preserves the MRE11 interaction domain, but loses a central region that includes several phosphorylation sites by ATM and ATR kinases, and the forkhead-associated and BRCA1 C-terminus domains. These lost regions have the functions of ATM activation, H2AX phosphorylation, MRN complex recruitment to damaged sites of DSBs, cell cycle arrest and DSB repair initiation (Rogers *et al*, 1997; Kobayashi *et al*, 2004; Czornak *et al*, 2008). Hence, a successful induction and expression of mutant NBS1 results in losing many functions related to DNA DSB repair. In this study, we showed that using Co-IP and western blot the mutant NBS1 retains the binding ability to MRE11 and competitively inhibits the binding of wild-type NBS1 to MRE11. Also, we showed the downregulation of wild-type NBS1 and RAD50 after Ad-NBS1 induction. These results are supposed to have occurred by the loss of NBS1 functions, such as H2AX phosphorylation or MRN complex recruitment to damaged sites of DSBs, and consequently induces a dominant-negative downregulation of MRN complex, possibly through inhibition of ATM activation and H2AX phosphorylation (Kobayashi *et al*, 2004; Czornak *et al*, 2008). These results support our hypothesis that mutant NBS1 disrupts MRN function. Indeed, our previous report indicated significantly increased cisplatin-induced DNA DSBs *in vitro* after mutant NBS1 transduction (Tran *et al*, 2004).

This study proved the benefit of the FGF2-targeting system, which has high transduction efficiency and enables a lower titre viral administration while still maintaining *in vitro* and *in vivo* tumour suppressive effect of cisplatin equivalent to a higher titre non-targeting transfer. Although adenovirus-mediated gene transfer is appealing, the infection of normal tissues and systemic toxicities, mainly caused by viral entrance into the bloodstream and viral trapping in the liver, limits the move into human clinical trials. To overcome these issues, the FGF2-targeted adenoviral system will be the most efficacious when applied to cells that have low CAR and high FGFRs expression. In our previous study, FGFR1 and FGFR2 expression was widely observed in both tumour cells and ECs of the human HNSCC xenograft model (Saito *et al*, 2009). Several studies have described a two- to 34-fold increase in intratumoral transduction efficiency (Rancourt *et al*, 1998; Doukas *et al*, 1999; Qin *et al*, 2005) and a two-fold increased transduction efficiency of adenoviral *TK* gene was observed for human HNSCC (Saito *et al*, 2009). Our results also support the benefit of FGF2 target with regard to intratumoral transduction efficiency.

In addition, our results showed that the number of apoptotic cells was not increased and microvessel density was decreased after FGF2-Ad-NBS1/cisplatin treatment, suggesting successful transduction into not only tumour cells, but also ECs by the FGF2-targeted adenovirus system. The FGF2-targeted system has been shown to induce highly efficient transduction in ECs that express relatively low levels of CAR receptor (Gupta *et al*, 2006; Saito *et al*, 2009). It has been reported that angiogenesis is enhanced and the expression levels of FGFR1 and FGFR2 are upregulated in cancer cells and ECs in HNSCC (Dellacono *et al*, 1997; Riedel *et al*, 2000). Although a direct effect in ECs as a result of mutant NBS1 gene transfer is supposed to be one mechanism for antiangiogenesis effect, downregulation of NF-*κ*B expression after FGF2-targeted Ad-NBS1 transduction that is seen in our study suggests other suppression mechanisms.

NF-*κ*B is a well-known transcription factor that is responsible for regulating many genes, including angiogenesis and apoptosis (Sun and Zhang, 2007). The NF-*κ*B transcription factor family is composed of p50, p52, RelA/p65, c-rel and Rel B. The homodimers and heterodimers are sequestered in the cytoplasm as an inactive form by the inhibitor of kappa B (I*κ*B). Upon stimulation, the I*κ*B kinase complex phosphorylates the *κ*B inhibitor, which then releases NF-*κ*B and allows its phosphorylation, nuclear translocation, binding and subsequent activation of target genes involved in the regulation of cell proliferation, survival, angiogenesis and metastasis (Brown *et al*, 1995). The inhibitor of NF-*κ*B had significant antitumour effects on oesophagus SCC by promoting apoptosis, and inhibiting proliferation and angiogenesis, as well as reduced the metastasis (Li *et al*, 2009). Furthermore, downregulation of NF-*κ*B might consolidate DNA damage and apoptosis induction because NF-*κ*B orchestrates a cell survival pathway together with the activation of cell cycle checkpoints and DNA repair (Janssens and Tschopp, 2006). However, it is not clear as to how NBS1 relates to the NF-*κ*B. Some reports indicate that when DSBs are generated by radiation, NBS-deficient cells exhibit a delayed and strongly reduced level of NF-*κ*B induction (Habraken and Piette, 2006) and one cascade for NF-*κ*B activation depends on ATM, which closely interacts with NBS1 (Habraken and Piette, 2006).

In this report, we have not shown the effect of FGF2-targeted Ad-NBS1 gene transduction to the PI3K/AKT/HIF angiogenesis pathway. However, many reports suggest that there is an interaction of NBS1 with key proteins involved in tumour angiogenesis induction pathway such as PI3K and HDM2 (Alt *et al*, 2005; Chen *et al*, 2008). We suppose that PI3K might be activated by NBS1, but might be inactivated by mutant NBS1. It has been reported that the reduced PI3K activity in NBS^−/−^ lymphoblasts is caused by an impaired expression of the SRC family kinases (Sagan *et al*, 2008). Similarly to our mutant NBS1, the NBS lymphoblastoid cell line used in this report expressed NBS1 with the common 657del5 mutation, which preserves PI3K activation domain. Therefore, our mutant NBS1 might have the potential to reduce PI3K activity and may result in the inactivation of the PI3K/AKT angiogenesis pathway. In fact, we have data with regard to the downregulation of HIF-1*α in vivo*, which is the major factor in PI3K/AKT pathway and regulates VEGF activation, after FGF2-targeted Ad-NBS1 gene transduction (data not shown). Downregulation of both Ras/MEK pathway and PI3K/AKT pathway may have synergistic effects in inducing tumour angiogenesis (Jiang and Liu, 2008), and with NF-*κ*B downregulation, a potent inhibition of tumour angiogenesis can be expected after FGF2-targeted Ad-NBS1 transduction.

Viral trapping in the liver is a major concern and an indicator for systemic toxicity in the systemic administration of adenoviral vector. It is reported that a majority of the adenoviral vector accumulates in the liver, which can cause severe liver toxicity, and that the liver is the most important organ to eliminate adenoviral vector genome through the innate immune system (Akiyama *et al*, 2004; Yao *et al*, 2010). Although the liver is the major site of adenoviral localisation owing to its high CAR expression, the FGF2-targeted adenoviral system can alter the distribution of the virus and deterge the viral vector from the liver. The amount of FGF2-targeted adenovirus delivery to the liver was shown as a 10- to 20-fold decrease, whereas an increase of transgene expression in the tumour tissue was observed after systemic administration (Printz *et al*, 2000). This decrease in liver deposition translates into a significant reduction in subsequent toxicity. In this study, a large systemic distribution of adenovirus was not expected because the virus was administered via intratumoral injection (Sewell *et al*, 1997). Nevertheless, the results confirmed adenovirus existence with high rates in the liver after Ad-NBS1 administration and absence of FGF2-targeted adenovirus. These results indicate that the FGF2-targeted adenoviral system results in a more efficient system with reduced viral titre application by its high transduction efficiency into cancer cells and with concomitant lower levels of liver toxicity.

This study proves the efficacy of Ad-NBS1 gene transduction *in vivo* to sensitise cisplatin chemotherapy by disrupting MRN function in DNA DSBs repair systems. Furthermore, our findings suggest that a more significant antitumour effect can be achieved through not only enhanced tumour cytotoxicity, but also antiangiogenetic effects when Ad-NBS1 is targeted to FGFRs. Concurrently, FGF2-targeted system can reduce liver toxicity by preventing adenoviral distribution. The great benefit of this strategy supports further clinical trials in the treatment of human HNSCC.

## Figures and Tables

**Table 1:** Distribution of Ad-NBS1 in the liver

	**PBS**	**Ad-NBS1**	**FGF2-Ad-NBS1**
Day 1	0/5	5/5	0/5
Day 3	0/5	4/5	0/5
Day 7	0/5	2/5	0/5
Day 14	0/5	2/5	0/5

Abbreviations: FGF2-Ad-NBS1=fibroblast growth factor 2-targeted adenoviral mutant Nijmegen breakage syndrome 1; PBS=phosphate-buffered saline.

## References

[bib1] Abuzeid WM, Jiang X, Shi G, Wang H, Paulson D, Araki K, Jungreis D, Carney J, O'Malley Jr BW, Li D (2009) Molecular disruption of RAD50 sensitizes human tumor cells to cisplatin-based chemotherapy. J Clin Invest 119: 1974–1985 19487811 10.1172/JCI33816 PMC2701852

[bib2] Akiyama M, Thorne S, Kirn D, Roelvink PW, Einfeld DA, King CR, Wickham TJ (2004) Ablating CAR and integrin binding in adenovirus vectors reduces nontarget organ transduction and permits sustained bloodstream persistence following intraperitoneal administration. Mol Ther 9: 218–230 14759806 10.1016/j.ymthe.2003.10.010

[bib3] Alt JR, Bouska A, Fernandez MR, Cerny RL, Xiao H, Eischen CM (2005) Mdm2 binds to Nbs1 at sites of DNA damage and regulates double strand break repair. J Biol Chem 280: 18771–18781 15734743 10.1074/jbc.M413387200

[bib4] Araki K, Ahmad SM, Li G, Bray Jr DA, Saito K, Wang D, Wirtz U, Sreedharan S, O'Malley Jr BW, Li D (2008) Retinoblastoma RB94 enhances radiation treatment of head and neck squamous cell carcinoma. Clin Cancer Res 14: 3514–3519 18519784 10.1158/1078-0432.CCR-07-4538

[bib5] Brown K, Gerstberger S, Carlson L, Franzoso G, Siebenlist U (1995) Control of I*κ*B-*α* proteolysis by site-specific, signal-induced phosphorylation. Science 267: 1485–1488 7878466 10.1126/science.7878466

[bib6] Chen YC, Chiang HY, Yang HM, Chen PM, Chang SY, Teng SC, Vanhaesebroeck B, Wu KJ (2008) Activation of phosphoinositide 3-kinase by the NBS1 DNA repair protein through a novel activation motif. J Mol Med 86: 401–412 18270679 10.1007/s00109-008-0302-x

[bib7] Chu G (1994) Cellular responses to cisplatin. The roles of DNA-binding proteins and DNA repair. J Biol Chem 269: 787–790 8288625

[bib8] Czornak K, Chughtai S, Chrzanowska KH (2008) Mystery of DNA repair: the role of the MRN complex and ATM kinase in DNA damage repair. J Appl Genet 49: 383–396 19029686 10.1007/BF03195638

[bib9] Dellacono FR, Spiro J, Eisma R, Kreutzer D (1997) Expression of basic fibroblast growth factor and its receptors by head and neck squamous carcinoma tumor and vascular endothelial cells. Am J Surg 174: 540–544 9374233 10.1016/s0002-9610(97)00169-4

[bib10] Doukas J, Hoganson DK, Ong M, Ying W, Lacey DL, Baird A, Pierce GF, Sosnowski BA (1999) Retargeted delivery of adenoviral vectors through fibroblast growth factor receptors involves unique cellular pathways. FASEB J 13: 1459–1466 10428769 10.1096/fasebj.13.11.1459

[bib11] Drummond JT, Anthoney A, Brown R, Modrich P (1996) Cisplatin and adriamycin resistance are associated with MutLalpha and mismatch repair deficiency in an ovarian tumor cell line. J Biol Chem 271: 19645–19648 8702663 10.1074/jbc.271.33.19645

[bib12] Fink D, Nebel S, Aebi S, Zheng H, Cenni B, Nehmé A, Christen RD, Howell SB (1996) The role of DNA mismatch repair in platinum drug resistance. Cancer Res 56: 4881–4886 8895738

[bib13] Goldman CK, Rogers BE, Douglas JT, Sosnowski BA, Ying W, Siegal GP, Baird A, Campain JA, Curiel DT (1997) Targeted gene delivery to Kaposi's sarcoma cells via the fibroblast growth factor receptor. Cancer Res 57: 1447–1451 9108444

[bib14] Gu DL, Gonzalez AM, Printz MA, Doukas J, Ying W, D'Andrea M, Hoganson DK, Curiel DT, Douglas JT, Sosnowski BA, Baird A, Aukerman SL, Pierce GF (1999) Fibroblast growth factor 2 retargeted adenovirus has redirected cellular tropism: evidence for reduced toxicity and enhanced antitumor activity in mice. Cancer Res 59: 2608–2614 10363982

[bib15] Gupta V, Wang W, Sosnowski S, Hofman FM, Chen TC (2006) Fibroblast growth factor-2-retargeted adenoviral vector for selective transduction of primary glioblastoma multiforme endothelial cells. Neurosurg Focus 20: E26 16709032

[bib16] Habraken Y, Piette J (2006) NF-kappaB activation by double-strand breaks. Biochem Pharmacol 72: 1132–1141 16965765 10.1016/j.bcp.2006.07.015

[bib17] Janssens S, Tschopp J (2006) Signals from within: the DNA-damage-induced NF-kappaB response. Cell Death Differ 13: 773–784 16410802 10.1038/sj.cdd.4401843

[bib18] Jiang BH, Liu LZ (2008) PI3K/PTEN signaling in tumorigenesis and angiogenesis. Biochim Biophys Acta 1784: 150–158 17964232 10.1016/j.bbapap.2007.09.008

[bib19] Kartalou M, Essigmann JM (2001) Mechanisms of resistance to cisplatin. Mutat Res 478: 23–43 11406167 10.1016/s0027-5107(01)00141-5

[bib20] Kelland L (2007) The resurgence of platinum-based cancer chemotherapy. Nat Rev Cancer 7: 573–584 17625587 10.1038/nrc2167

[bib21] Kleeff J, Fukahi K, Lopez ME, Friess H, Büchler MW, Sosnowski BA, Korc M (2002) Targeting of suicide gene delivery in pancreatic cancer cells via FGF receptors. Cancer Gene Ther 9: 522–532 12032663 10.1038/sj.cgt.7700464

[bib22] Kobayashi J, Antoccia A, Tauchi H, Matsuura S, Komatsu K (2004) NBS1 and its functional role in the DNA damage response. DNA Repair 3: 855–861 15279770 10.1016/j.dnarep.2004.03.023

[bib23] Li B, Li YY, Tsao SW, Cheung ALM (2009) Targeting NF-*κ*B signaling pathway suppresses tumor growth, angiogenesis, and metastasis of human esophageal cancer. Mol Cancer Ther 8: 2635–2644 19723887 10.1158/1535-7163.MCT-09-0162

[bib24] Li D, Day KV, Yu S, Shi G, Liu S, Guo M, Xu Y, Sreedharan S, O'Malley Jr BW (2002) The role of adenovirus-mediated retinoblastoma 94 in the treatment of head and neck cancer. Cancer Res 62: 4637–4644 12183420

[bib25] Li D, Duan L, Freimuth P, O'Malley Jr BW (1999) Variability of adenovirus receptor density influences gene transfer efficiency and therapeutic response in head and neck cancer. Clin Cancer Res 5: 4175–4181 10632357

[bib26] Martin LP, Hamilton TC, Schilder RJ (2008) Platinum resistance: the role of DNA repair pathways. Clin Cancer Res 14: 1291–1295 18316546 10.1158/1078-0432.CCR-07-2238

[bib27] O'Malley Jr BW, Li D, Carney J, Rhee J, Suntharalingam M (2003) Molecular disruption of the MRN(95) complex induces radiation sensitivity in head and neck cancer. Laryngoscope 113: 1588–1594 12972939 10.1097/00005537-200309000-00034

[bib28] Perez RP (1998) Cellular and molecular determinants of cisplatin resistance. Eur J Cancer 34: 1535–1542 9893624 10.1016/s0959-8049(98)00227-5

[bib29] Printz MA, Gonzalez AM, Cunningham M, Gu DL, Ong M, Pierce GF, Aukerman SL (2000) Fibroblast growth factor 2-retargeted adenoviral vectors exhibit a modified biolocalization pattern and display reduced toxicity relative to native adenoviral vectors. Hum Gene Ther 11: 191–204 10646650 10.1089/10430340050016265

[bib30] Qin M, Escuadro B, Sharma S, Batra RK (2005) Gene transfer mediated by native versus fibroblast growth factor-retargeted adenoviral vectors into lung cancer cells. Am J Respir Cell Mol Biol 32: 211–217 15626775 10.1165/rcmb.2004-0226OC

[bib31] Rancourt C, Rogers BE, Sosnowski BA, Wang M, Piché A, Pierce GF, Alvarez RD, Siegal GP, Douglas JT, Curiel DT (1998) Basic fibroblast growth factor enhancement of adenovirus-mediated delivery of the herpes simplex virus thymidine kinase gene results in augmented therapeutic benefit in a murine model of ovarian cancer. Clin Cancer Res 4: 2455–2461 9796978

[bib32] Riedel F, Götte K, Bergler W, Rojas W, Hörmann K (2000) Expression of basic fibroblast growth factor protein and its down-regulation by interferons in head and neck cancer. Head Neck 22: 183–189 10679908 10.1002/(sici)1097-0347(200003)22:2<183::aid-hed11>3.0.co;2-r

[bib33] Rogers BE, Douglas JT, Ahlem C, Buchsbaum DJ, Frincke J, Curiel DT (1997) Use of a novel cross-linking method to modify adenovirus tropism. Gene Ther 4: 1387–1392 9472563 10.1038/sj.gt.3300541

[bib34] Sagan D, Eckardt-Schupp F, Eichholtz-Wirth H (2008) Reduced expression of SRC family kinases decreases PI3K activity in NBS1−/− lymphoblasts. Biochem Biophys Res Commun 377: 181–186 18835245 10.1016/j.bbrc.2008.09.098

[bib35] Saito K, Khan K, Sosnowski B, Li D, O'Malley BW (2009) Cytotoxicity and antiangiogenesis by fibroblast growth factor 2-targeted Ad-TK cancer gene therapy. Laryngoscope 119: 665–674 19213040 10.1002/lary.20127

[bib36] Sewell DA, Li D, Duan L, Westra WH, O'Malley Jr BW (1997) Safety of *in vivo* adenovirus-mediated thymidine kinase treatment of oral cancer. Arch Otolaryngol Head Neck Surg 123: 1298–1302 9413357 10.1001/archotol.1997.01900120048007

[bib37] Sun XF, Zhang H (2007) NFKB and NFKBI polymorphisms in relation to susceptibility of tumour and other diseases. Histol Histopathol 22: 1387–1398 17701919 10.14670/HH-22.1387

[bib38] Tran HM, Shi G, Li G, Carney JP, O'Malley Jr BW, Li D (2004) Mutant Nbs1 enhances cisplatin-induced DNA damage and cytotoxicity in head and neck cancer. Otolaryngol Head Neck Surg 131: 477–484 15467621 10.1016/j.otohns.2004.04.019

[bib39] Varon R, Vissinga C, Platzer M, Cerosaletti KM, Chrzanowska KH, Saar K, Beckmann G, Seemanová E, Cooper PR, Nowak NJ, Stumm M, Weemaes CM, Gatti RA, Wilson RK, Digweed M, Rosenthal A, Sperling K, Concannon P, Reis A (1998) Nibrin, a novel DNA double-strand break repair protein, is mutated in Nijmegen breakage syndrome. Cell 93: 467–476 9590180 10.1016/s0092-8674(00)81174-5

[bib40] Wakulich C, Jackson-Boeters L, Daley TD, Wysocki GP (2002) Immunohistochemical localization of growth factors fibroblast growth factor-1 and fibroblast growth factor-2 and receptors fibroblast growth factor receptor-2 and fibroblast growth factor receptor-3 in normal oral epithelium, epithelial dysplasias, and squamous cell carcinoma. Oral Surg Oral Med Oral Pathol Oral Radiol Endod 93: 573–579 12075207 10.1067/moe.2002.124461

[bib41] Weidner N (1995) Current pathologic methods for measuring intratumoral microvessel density within breast carcinoma and other solid tumors. Breast Cancer Res Treat 36: 169–180 8534865 10.1007/BF00666038

[bib42] Williams RS, Williams JS, Tainer JA (2007) Mre11-Rad50-Nbs1 is a keystone complex connecting DNA repair machinery, double-strand break signaling, and the chromatin template. Biochem Cell Biol 85: 509–520 17713585 10.1139/O07-069

[bib43] Yao X, Yoshioka Y, Morishige T (2010) Adenovirus vector covalently conjugated to polyethylene glycol with a cancer-specific promoter suppresses the tumor growth through systemic administration. Biol Pharm Bull 33: 1073–1076 20522982 10.1248/bpb.33.1073

